# The Effects of Propofol or Dexmedetomidine Sedation on Postoperative Recovery in Elderly Patients Receiving Lower Limb Surgery under Spinal Anesthesia: A Retrospective Propensity Score-Matched Analysis

**DOI:** 10.3390/jcm10010135

**Published:** 2021-01-03

**Authors:** Jin-Woo Park, Eun-Kyoung Kim, Hun-Taek Lee, Seongjoo Park, Sang-Hwan Do

**Affiliations:** 1 Department of Anesthesiology and Pain Medicine, Seoul National University Bundang Hospital, Seongnam 13620, Korea; jinul8282@gmail.com (J.-W.P.); htlee0@gmail.com (H.-T.L.); struka@snubh.org (S.P.); 2 Department of Anesthesiology and Pain Medicine, Noh Jong Hoon Plastic Surgery, Seoul 06030, Korea; eunkyoung2lovely@gmail.com; 3 Department of Anesthesiology and Pain Medicine, College of Medicine, Seoul National University, Seoul 03080, Korea

**Keywords:** dexmedetomidine, intraoperative sedation, postoperative delirium, propofol

## Abstract

Propofol and dexmedetomidine are the two most popular intravenous sedatives during anesthesia. However, data comparing the effects of these two sedatives during spinal anesthesia on postoperative recovery are still insufficient. We retrospectively analyzed the medical records of patients aged ≥65 years who underwent orthopedic surgery under spinal anesthesia between March 2012 and February 2017. The patients were allocated into two groups according to the intraoperative sedatives: the propofol group and dexmedetomidine group. We analyzed the incidence of postoperative delirium, analgesic requirement, and rescue anti-emetic treatment. A total of 1045 patients were included in the analysis. After propensity score matching with the propofol group, the dexmedetomidine group showed a lower incidence of postoperative delirium (odds ratio, 0.19; 95% CI, 0.07–0.56; *p* = 0.011). Postoperative analgesic and anti-emetic requirement were not significantly different between the two groups (*p* = 0.156 and 0.245, respectively). Multivariate logistic regression analysis revealed that intraoperative sedation, age, preoperative albumin level, and hip surgery were significantly associated with the incidence of postoperative delirium. This study showed that intraoperative dexmedetomidine sedation under spinal anesthesia during lower limb surgery is associated with a lower incidence of postoperative delirium compared with propofol sedation.

## 1. Introduction

In elderly patients, fractures or degenerative diseases of the lower extremities are a common occurrence. In such cases, orthopedic surgeries are actively performed. To relieve anxiety and stress during lower limb surgeries, intravenous (IV) sedation under regional anesthesia is often required [1,2].

Propofol and dexmedetomidine are the two most popular IV sedatives. Propofol has fast onset and offset of action, and its target-controlled infusion allows easy titration and adequate sedation [3]. Dexmedetomidine is an α-2 agonist acting on adrenoreceptors in different tissues, and it induces electroencephalographic activity similar to natural sleep without affecting the ventilatory response to carbon dioxide [4]. Dexmedetomidine has been reported to reduce the incidence of postoperative delirium (POD) [5,6]. It was also demonstrated that intraoperative dexmedetomidine treatment is associated with decreased postoperative pain and a lower incidence of nausea and vomiting [7,8,9]. However, the above effects were not consistent among different studies [10,11,12], and propofol is known to reduce postoperative analgesic and anti-emetic usage [13,14]. Data comparing the effects between dexmedetomidine and propofol sedation during spinal anesthesia on postoperative recovery are still insufficient, especially in surgical settings.

In this retrospective study, we aimed to analyze the incidence of POD, postoperative analgesia, and anti-emetic requirement between elderly patients treated with propofol sedation and those treated with dexmedetomidine sedation during lower limb surgery under spinal anesthesia.

## 2. Materials and Methods

This retrospective observational study was conducted in a single tertiary academic hospital after receiving approval from the Institutional Review Board (IRB) of Seoul National University Bundang Hospital (IRB Number: B-1909/564-104; approval date: August 26, 2019). The requirement for informed consent was waived by the IRB due to the retrospective nature of this study.

### 2.1. Study Population

The medical records of patients ≥65 years who underwent orthopedic surgery under spinal anesthesia at Seoul National University Bundang Hospital between March 1, 2012 and February 28, 2017 were reviewed. The patients who received general or epidural anesthesia, those who needed postoperative intensive care unit (ICU) admission owing to major adverse events during surgery, those with preoperative medication within 24 hours prior to surgery affecting postoperative nausea and vomiting (anti-emetics, steroids, or antihistamine), those with preoperative dementia, and those with an incomplete medical record were excluded from the analysis. The patients who did not receive intraoperative IV sedation with propofol or dexmedetomidine, as well as those who underwent minor surgery with a hospital stay of less than 3 postoperative days, were also excluded. If a patient underwent multiple surgeries during the study period, the first inclusive case was solely indicated.

### 2.2. Spinal Anesthesia and Intraoperative Sedation

The patients routinely received IV midazolam premedication at the preoperative holding area. In our hospital, an optimal dose of 0.5% hyperbaric bupivacaine or 0.75% levobupivacaine and 10–20 μg fentanyl was injected intrathecally for the induction of spinal anesthesia. After confirmation of proper spinal block height and hemodynamic stability, the patients were asked whether they wanted to be sedated. If the patient wanted sedation during surgery, IV propofol or dexmedetomidine was used at the discretion of the anesthesiologist in the following manner: (1) propofol sedation, target-controlled infusion of propofol using an Orchestra infusion pump system (Fresenius vial, Brezins, France) within an effect-site concentration of 0.5–2.0 μg·mL^−1^; (2) dexmedetomidine sedation, loading a dose with 1 μg·kg^−1^ dexmedetomidine over 10 min, and then continuous infusion at 0.1–0.5 μg·kg^−1^·h^−1^. The infusion rate or effect-site concentration was adjusted to achieve a modified Observer’s Assessment of Alertness/Sedation scale of 3 or 4. The modified Observer’s Assessment of Alertness/Sedation scale is graded as follows: responds readily to name spoken in normal tone (5); lethargic response to name spoken in normal tone (4); responds only after name is called loudly and/or repeatedly (3); responds only after mild prodding or shaking (2); does not respond to mild prodding or shaking (1) [15].

### 2.3. Study Outcomes

When patients showed abnormal behavior at the ward during the postoperative period, such as agitation, anxiety, depression, fear, insomnia, and hallucinations, orthopedists sought neuropsychiatric consultation. Upon request, a psychiatrist examined the patient immediately and determined POD using the Confusion Assessment Method for the intensive care unit (CAM-ICU) [16]. The incidence of POD was the primary outcome of this retrospective study. Postoperative abnormal behavior needing neuropsychiatric consultation, the amount of opioid for postoperative analgesia (aggregated and calculated as a morphine equivalent dose), and the number of anti-emetic treatments during 72 hours after surgery were also assessed. Demographic data (age, sex, and body mass index), preoperative patient condition (American Society of Anesthesiologists (ASA) classification, hypertension, diabetes mellitus, ischemic heart disease, cerebrovascular disease, anemia, and albumin level), the Charlson comorbidity index (CCI), type of surgery, and operational data (operation time, estimated blood loss, intraoperative blood pressure, perioperative red blood cell transfusion, admission period, years at surgery, and midazolam premedication) were collected. We investigated the variables expected to affect postoperative recovery. 

### 2.4. Statistical Analysis

The patients were allocated to two groups based on the sedatives used during spinal anesthesia: the propofol group and dexmedetomidine group. Continuous variables are presented as median with interquartile ranges, and categorical variables are shown as numbers (%). The Mann–Whitney *U* test was used to compare continuous variables, and the chi-square or Fisher’s exact test was used to compare categorical variables, as appropriate, between the two groups. 

Propensity score matching was performed to minimize the risk of confounder effects and to equalize potential prognostic factors between the two groups. The patients were matched at a 1:1 ratio by the nearest-neighbor method without replacement (caliper = 0.1). All covariates of demographics, comorbidities, anesthetic characteristics, and operation data were included in the propensity matching model, and the propensity score was calculated with a logistic regression analysis. The standardized mean difference was tested for each covariate at <0.1 for the balance between the groups.

To analyze the association between each variable and POD, we performed a univariate logistic regression among the cohort patients. The variables with *p* < 0.2 from the univariate regression were included in the final multivariate logistic regression analysis. For all statistical analyses, we used SPSS version 21.0 software (SPSS Inc., IBM, Chicago, IL, USA). A two-sided *p*-value of <0.05 was considered statistically significant.

## 3. Results

**Figure 1:**
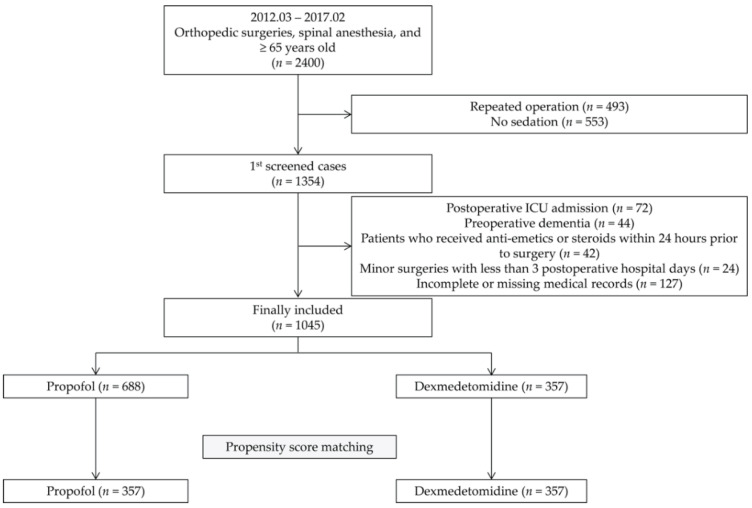
Flow chart of patient selection. ICU, intensive care unit.

A total of 2400 patients underwent orthopedic surgeries from March 2012 to February 2017. Among them, 1045 patients were finally included in the analysis (propofol group, 688; dexmedetomidine group, 357). After propensity score matching, 357 patients were allocated to each group (Figure 1).

Patient characteristics, preoperative conditions, and information about surgery and anesthesia, before and after the propensity score matching, are shown in Table 1. All covariates showed standardized mean differences less than 0.1 after matching, indicating a good balance. Of the original 1045 patients, the incidence of POD was significantly lower in the dexmedetomidine group than in the propofol group (odds ratio, 0.19; 95% CI, 0.07~0.55; *p* = 0.007; Table 2). Postoperative requirements for opioid analgesics and anti-emetics were not significantly different between the two.

The results of univariate and multivariate logistic regression for POD are presented in Table 3 and Table 4, respectively. According to the final multivariate regression, intraoperative dexmedetomidine sedation (odds ratio, 0.33; 95% CI, 0.14~0.77; *p* = 0.011), age of patients (odds ratio, 1.10; 95% CI, 1.05~1.15; *p* < 0.001), preoperative albumin level (odds ratio, 0.40; 95% CI, 0.18~0.99; *p* = 0.043), and hip surgery (odds ratio, 2.86; 95% CI, 1.45~5.65; *p* = 0.002) were significantly related to the incidence of POD (Table 4). The multivariate logistic regression showed a proper goodness of fit, assessed with the Hosmer–Lemeshow test (*p* = 0.667).

## 4. Discussion

POD is one of the most prevalent and deleterious postoperative complications in geriatric patients. It is known to be associated with longer hospitalization, delayed rehabilitation, prolonged cognitive dysfunction, and increased mortality [17,18,19,20]. Compared with propofol, dexmedetomidine sedation during ICU care is known to reduce the incidence and duration of POD in post-cardiac surgery [5]. Intraoperative dexmedetomidine treatment has also been reported to decrease POD incidence in post-joint replacements operations [21]. However, most previous studies performed the sedation at the ICU or additionally infused dexmedetomidine during general anesthesia [5,21,22]. A previous retrospective observational study reported that IV sedation with dexmedetomidine during spinal anesthesia, compared with propofol sedation, had a beneficial effect in reducing agitated behavior, which might be consistent with our finding [23]. However, this previous retrospective study did not use CAM-ICU, a standard method of POD diagnosis; instead, the patients’ medical records were reviewed for signs of agitated behavior. To the best of our knowledge, the present study is the first to confirm the effect of dexmedetomidine and propofol sedation during spinal anesthesia on the incidence of POD based on accurate criteria (CAM-ICU).

Several risk factors are known to be related to POD: older age, preoperative cognitive dysfunction, hypoalbuminemia, and pre-existing comorbidities [18,19,24,25]. POD also occurs frequently in certain types of surgeries, including hip surgery and major cardiac surgery [19,26]. In this retrospective study, we were unable to assess the preoperative cognitive status of patients since the cognitive function test is not routinely performed preoperatively in our hospital. Rather, we excluded patients with preoperative dementia from the analysis to minimize the effect of cognitive dysfunction before surgery on POD. Hypoalbuminemia, age, and hip surgery were found to be associated with the incidence of POD in our analysis, which is in line with previous publications [18,19,24,25,26].

In previous studies, ASA ≥ 3 and higher Charlson index were reported to be risk factors of postoperative abnormal behavior [18,23,24,27]. However, in our regression model, both these factors were not significantly related to the incidence of POD. Based on ASA and CCI, our patients generally showed similar comorbidity levels. For example, only 8.6% of the cohort in our analysis had an ASA status of ≥3, which might make it difficult to show statistical differences in POD incidence according to the status of comorbidities. Benzodiazepine use is also known to precipitate delirium through its effect on gamma-aminobutyric acid. This phenomenon occurs when it is given continuously rather than intermittently [28,29]. In our study, midazolam was premedicated by a single injection of small dose, which would not significantly affect the incidence of POD.

Due to the retrospective nature of this study, we were unable to directly assess the postoperative pain level in our patients. Conversely, we retrospectively investigated the opioid analgesics consumed postoperatively. A few studies reported that dexmedetomidine sedation during regional anesthesia reduced postoperative pain and postoperative analgesic requirement, compared with propofol sedation [7,30]. The data comparing the effects of these two sedatives during regional anesthesia on postoperative pain management are very limited. In our study, postoperative opioid consumption was not significantly different between the two groups. However, perioperative pain management was not controlled in our study. According to previous studies, both dexmedetomidine and propofol are helpful in the improvement of postoperative analgesia [5,6,31,32]. Therefore, strictly controlled analgesic management should be necessary to show any statistical differences between these two sedation methods. Likewise, the requirement of anti-emetics was comparable between the dexmedetomidine group and the propofol group. Both propofol and dexmedetomidine have been reported to reduce postoperative nausea and vomiting [8,33,34], which are highly affected by opioid treatment. Therefore, tightly controlled prospective studies should be performed to confirm the analgesic and anti-emetic effects of the two most popular sedatives during spinal anesthesia.

This study has some limitations. First, the patients’ cognitive status could not be directly evaluated. Hypoactive delirium or mixed-type delirium could often not be recognized in our retrospective investigation because the patients were formally assessed for POD when they outwardly manifested abnormal behavior. The incidence of POD might be underestimated. Some patients with preoperative cognitive dysfunction were included, although we excluded patients diagnosed with dementia. Prospective studies with preoperative and postoperative cognitive assessments are required to overcome these issues. Second, given the retrospective nature of this study, the intraoperative sedation during spinal anesthesia was not randomized. To minimize selection bias, we tried to evenly balance the characteristics between the two study groups using propensity score matching. Third, the etiology of postoperative delirium is very complex; there might be unknown confounders affecting our analysis. Fourth, we investigated the analgesic opioid consumption as an indicator of postoperative pain management. Nonsteroidal anti-inflammatory drugs were also used for rescue analgesia in a subset of the patient population, not for patient-controlled analgesia. However, nonsteroidal anti-inflammatory drugs accounted for a relatively small portion of postoperative analgesia, and opioids could be summarized in morphine-equivalent dose. Finally, the generalizability of our study findings may be limited due to the retrospective analysis of data from a single medical center.

In conclusion, this retrospective study demonstrated that intraoperative dexmedetomidine sedation during lower limb surgery under spinal anesthesia is associated with a lower incidence of postoperative delirium in elderly patients, compared with propofol sedation. However, postoperative opioid consumption and the requirement for anti-emetics were not significantly different between the two sedative groups. This finding is clinically valuable because it provides important information about the effects of commonly used IV sedatives during spinal anesthesia on postoperative recovery in elderly patients. Our findings should be confirmed in future prospective studies.

## Figures and Tables

**Table 1:** Baseline characteristics before and after propensity score matching.

	Unmatched Cohort (*n* = 1045)				Matched Cohort (*n* = 714)			
	Propofol *n* = 688	DMED *n* = 357	SMD	*p*	Propofol *n* = 357	DMED *n* = 357	SMD	*p*
Age, year	74.0 (70.0–79.0)	74.0 (70.0–79.0)	0.004	0.870	74.0 (70.0–78.0)	74.0 (70.0–79.0)	0.018	0.954
Sex								
Male	123 (17.9)	68 (19.0)	0.030	0.643	68 (19.0)	68 (19.0)	< 0.001	> 0.999
Female	565 (82.1)	289 (81.0)			289 (81.0)	289 (81.0)		
BMI, kg m^–2^	25.6 (23.2–28.0)	25.6 (23.3–28.2)	0.088	0.580	25.7 (23.4–28.1)	25.6 (23.3–28.2)	0.047	0.951
ASA status (I/II)								
I	85 (12.4)	44 (12.3)	0.019	0.958	40 (11.2)	44 (12.3)	0.038	0.879
II	545 (79.2)	281 (78.7)			283 (79.3)	281 (78.7)		
III	58 (8.4)	32 (9.0)			34 (9.5)	32 (9.0)		
Hypertension	384 (55.8)	205 (57.4)	0.032	0.619	203 (56.9)	205 (57.4)	0.011	0.880
Diabetes mellitus	163 (23.7)	84 (23.5)	0.004	0.953	76 (21.3)	84 (23.5)	0.054	0.473
Ischemic heart disease	58 (8.4)	26 (7.3)	0.043	0.518	24 (6.7)	26 (7.3)	0.022	0.769
Cerebrovascular disease	47 (6.8)	31 (8.7)	0.069	0.280	30 (8.4)	31 (8.7)	0.010	0.893
CCI	0.0 (0.0–1.0)	0.0 (0.0–1.0)	0.003	0.804	0.0 (0.0–1.0)	0.0 (0.0–1.0)	0.018	0.574
Anemia (Hb < 10 g dL^–1^)	44 (6.4)	16 (4.5)	0.084	0.207	13 (3.6)	16 (4.5)	0.043	0.570
Albumin, g dL^–1^	4.2 (4.0–4.4)	4.3 (4.0–4.4)	0.055	0.555	4.3 (4.1–4.4)	4.3 (4.0–4.4)	0.075	0.462
Type of surgery								
CRIF								
Femur	18 (2.6)	2 (0.6)	0.233	0.081	2 (0.6)	2 (0.6)	0.055	0.881
Tibia, fibula, and foot	2 (0.3)	1 (0.3)			2 (0.6)	1 (0.3)		
ORIF								
Femur	56 (8.1)	21 (5.9)			23 (6.4)	21 (5.9)		
Tibia, fibula, and foot	28 (4.1)	7 (2.0)			8 (2.2)	7 (2.0)		
Replacement								
Hip	124 (18.0)	72 (20.2)			73 (20.4)	72 (20.2)		
Knee	426 (61.9)	237 (66.4)			240 (67.2)	237 (66.4)		
Ankle	4 (0.6)	3 (0.8)			1 (0.3)	3 (0.8)		
Others	30 (4.4)	14 (3.9)			8 (2.2)	14 (3.9)		
Operative characteristics								
Operation time (min)	135.0 (115.0–150.0)	135.0 (120.0–150.0)	0.062	0.304	135.0 (120.0–150.0)	135.0 (120.0–150.0)	0.021	0.922
Estimated blood loss (mL)	100.0 (50.0–200.0)	100.0 (50.0–200.0)	0.014	0.195	100.0 (50.0–200.0)	100.0 (50.0–200.0)	< 0.001	0.980
MBP, mmHg	72.9 (68.5–78.3)	73.7 (68.7–78.9)	0.019	0.333	73.0 (68.3–78.3)	73.7 (68.7–78.9)	0.023	0.415
RBC transfusion	135 (19.6)	64 (17.9)	0.043	0.508	67 (18.8)	64 (17.9)	0.022	0.772
Admission period	7.5 (5.4–12.5)	7.4 (5.4–12.4)	0.041	0.622	7.4 (5.4–12.4)	7.4 (5.4–12.4)	0.065	0.566
Years at surgery								
2012–2013	187 (27.2)	96 (26.9)	0.058	0.347	92 (25.8)	96 (26.9)	0.042	0.316
2014–2015	315 (45.8)	150 (42.0)			169 (47.3)	150 (42.0)		
2016–2017	186 (27.0)	111 (31.1)			96 (26.9)	111 (31.1)		
Premedication								
Midazolam (mg)	3.0 (1.0–3.0)	3.0 (1.0–3.0)	0.035	0.571	3.0 (1.0–3.0)	3.0 (1.0–3.0)	0.041	0.684

Abbreviations: DMED, dexmedetomidine; SMD, standardized mean difference; BMI, body mass index; ASA, American Society of Anesthesiologists; CCI, Charlson comorbidity index; CRIF, closed reduction of fracture with internal fixation; ORIF, open reduction of fracture with internal fixation; MBP, mean blood pressure; RBC, red blood cell. Groups (*p* = 0.071 and 0.527, respectively; Table 2). After propensity score matching, similar results were obtained. The dexmedetomidine group showed a lower incidence of POD (odds ratio, 0.19; 95% CI, 0.07~0.56; *p* = 0.011). Nonetheless, postoperative opioid and anti-emetic consumption was comparable between the two groups (*p* = 0.156 and 0.245, respectively; Table 2).

**Table 2:** POD, postoperative analgesia, and anti-emetic treatment from postoperative day 0 to 3, before and after propensity score matching.

	Propofol	DMED	Odds Ratio (95% CI)	*p*
Before matching				
POD	38/688 (5.5)	7/357 (2.0)	0.19 (0.07–0.55)	0.007
Neuropsychiatry consultation	51/688 (7.4)	12/357 (3.4)	0.43 (0.23–0.83)	0.009
MEC, mg	126.0 (69.9–207.3)	123.0 (57.0–195.0)		0.071
NRA	1.0 (0.0–2.0)	1.0 (0.0–3.0)		0.527
After matching				
POD	20/357 (5.6)	7/357 (2.0)	0.19 (0.07–0.56)	0.011
Neuropsychiatry consultation	26/357 (7.3)	12/357 (3.4)	0.44 (0.22–0.90)	0.020
MEC, mg	119.0 (70.0–202.0)	123.0 (57.0–195.0)		0.156
NRA	1.0 (0.0–2.0)	1.0 (0.0–3.0)		0.245

Abbreviations: POD, postoperative delirium; DMED, dexmedetomidine; MEC, morphine equivalent consumption; NRA, number of rescue anti-emetic treatment.

**Table 3:** Results of univariate analysis of variables associated with POD in the 1045 cohort of patients.

	Odds Ratio (95% CI)	*p*
Sedative		
Propofol	1	
DMED	0.34 (0.15–0.77)	0.010
Age	1.15 (1.10–1.20)	<0.001
Sex		
Male	1	
Female	0.77 (0.38–1.59)	0.485
BMI	0.86 (0.80–0.93)	<0.001
ASA		
I	1	
II	5.89 (0.80–43.31)	0.082
III	12.59 (1.55–102.49)	0.018
Hypertension	1.06 (0.58–1.94)	0.845
Diabetes mellitus	1.18 (0.60–2.33)	0.625
Ischemic heart disease	1.46 (0.56–3.80)	0.441
Cerebrovascular disease	0.82 (0.29– 2.35)	0.711
CCI	1.28 (1.05–1.56)	0.016
Anemia	4.65 (2.13–10.18)	<0.001
Albumin	0.33 (0.19–0.57)	<0.001
Hip surgery	4.60 (2.49–8.49)	<0.001
Operation time	0.99 (0.98–1.00)	0.006
Estimated blood loss	1.00 (1.00–1.00)	0.381
MBP	1.01 (0.98–1.05)	0.442
RBC transfusion	3.02 (1.63–5.60)	<0.001
Years at surgery		
2012-2013	1	
2014-2015	0.95 (0.48–1.90)	0.894
2016-2017	0.60 (0.26–1.41)	0.242
Midazolam	0.67 (0.51–0.87)	0.003
MEC	1.00 (1.00–1.00)	0.577
NRA	1.17 (0.99–1.39)	0.063

Abbreviations: POD, postoperative delirium; DMED, dexmedetomidine; BMI, body mass index; ASA, American Society of Anesthesiologists; CCI, Charlson comorbidity index; MBP, mean blood pressure; RBC, red blood cell; MEC, morphine equivalent consumption; NRA, number of rescue anti-emetic treatment.

**Table 4:** Results of the multivariate analysis of variables associated with POD.

	Odds Ratio (95% CI)	*p*
Sedative		
Propofol	1	
DMED	0.33 (0.14–0.77)	0.011
Age	1.10 (1.05–1.15)	<0.001
BMI	0.99 (0.90–1.08)	0.769
ASA		
I	1	
II	5.43 (0.72–40.70)	0.100
III	6.32 (0.74–54.10)	0.093
CCI	1.07 (0.84–1.37)	0.597
Anemia	2.22 (0.94–5.24)	0.070
Albumin	0.43 (0.23–0.98)	0.046
Hip surgery	2.86 (1.45–5.65)	0.002
Operation time	1.00 (0.99–1.01)	0.691
RBC transfusion	1.17 (0.53–2.56)	0.698
Midazolam	1.04 (0.76–1.41)	0.818
NRA	1.02 (0.80–1.31)	0.856

Abbreviations: POD, postoperative delirium; DMED, dexmedetomidine; BMI, body mass index; ASA, American Society of Anesthesiologists; MBP, mean blood pressure; NRA, number of rescue anti-emetic treatment.

## Data Availability

The datasets generated and analyzed during the current study are available on request to the corresponding author on reasonable request.
